# Outcomes of an adapted prolonged exposure psychotherapy for people with early phase psychosis, substance misuse, and a history of adversity: the PE + trial

**DOI:** 10.1186/s12888-024-06050-1

**Published:** 2024-10-14

**Authors:** Victoria C Patterson, Philip G Tibbo, Sherry H Stewart, Joel Town, Candice E Crocker, Zenovia Ursuliak, Siranda Lee, Jason Morrison, Sabina Abidi, Kara Dempster, Maria Alexiadis, Neal Henderson, Alissa Pencer

**Affiliations:** 1 Department of Psychology & Neuroscience, Dalhousie University, Halifax, NS Canada; 2 Department of Psychiatry, Dalhousie University, Halifax, NS Canada; 3 Mental Health and Addictions, Nova Scotia Health, Halifax, NS Canada; 4 Mental Health and Addictions, IWK Health, Halifax, NS Canada

**Keywords:** Prolonged exposure, Early phase psychosis, Adversity, Substance misuse, Cognitive-behavioural therapy

## Abstract

**Background:**

Several adversity-focused treatment trials have reported improvements to adversity sequelae (e.g., PTSD symptoms) and decreases in psychotic symptoms among individuals with psychotic disorders. Yet, no trials have examined the impact of adversity-focused treatment on substance use or examined the outcomes among an early phase psychosis population. These gaps in both the research literature and clinical practice have resulted in less knowledge about the outcomes of adversity-focused treatment at this stage of illness, including the impact on substance use.

**Methods:**

The outcomes of an adapted prolonged exposure protocol (PE+) among an early phase psychosis population were examined using a multiple-baseline design. Nineteen adults with a psychotic disorder, current substance misuse, and a history of adversity were recruited from an early psychosis program. Participants were randomized to treatment start time and participated in a 15-session course of PE + therapy. Ten assessments were completed focusing on primary outcomes (i.e., adversity sequelae, negative psychotic symptoms, substance misuse) and secondary outcomes (i.e., functioning, hopelessness, experiential avoidance). The Reliable Change Index (RCI) was used to establish whether there were clinically significant changes to primary or secondary outcomes.

**Results:**

Half or more of treatment completers experienced clinically significant changes to most domains of adversity sequelae, no participants experienced improvements in negative psychotic symptoms, and substance misuse increased for several participants. In terms of secondary outcomes, functioning and experiential avoidance were improved for a number of participants, while hopelessness decreased for only one participant. Participants reported high satisfaction with the PE + treatment, and exposure and coping skills were rated as the most helpful elements of treatment.

**Conclusions:**

Reductions in adversity sequelae were observed following PE + treatment, suggesting that adversity-focused treatment may be beneficial for an early psychosis population. Yet, few positive changes to psychotic symptoms or substance use were observed. Further integrating treatment strategies for psychosis and substance use into PE + may be required to effectively treat the links between psychosis, adversity sequelae, and substance use. Future studies should make efforts to integrate substance use strategies into adversity treatment trials for people with psychotic disorders.

**Trial registration:**

Clinicaltrials.gov, NCT04546178; registration posted 11/09/2020, https://clinicaltrials.gov/ct2/show/NCT04546178?term=NCT04546178&draw=2&rank=1.

## Introduction

Previous studies have found more negative outcomes for those individuals with psychotic disorders (PDs) and a history of adverse events (AEs), both in terms of course of illness (e.g., more distressing hallucinations, greater risk of suicide) [1, 2] and treatment outcomes (e.g., fewer treatment goals met, slower improvement) [3, 4]. In addition, several studies have suggested that psychosis itself can function as an AE for individuals with PDs [5–8], resulting in similarly poor outcomes. Due to the significant impacts of AEs on illness course and treatment, there has been a consistent call in the literature to develop adversity-focused interventions for people with PDs and a history of AEs to improve outcomes for this group [9–11]. However, substance misuse (SM) is a very common comorbid issue for people with PDs and a history of AEs, with quite elevated prevalence rates [12]. Yet, SM is rarely considered in the presence of AEs, despite its similarly deleterious impact on psychotic symptoms and outcomes of treatment [13] and its relationship with AEs sequelae (e.g., PTSD symptoms, depressive symptoms). Taken together, the above literature, along with recent preliminary clinical guidelines for adversity-focused treatments for people with PDs [14], suggest the need for an integrated psychological treatment capable of simultaneously addressing AE sequelae, SM, and psychotic symptoms.

Several studies have examined the efficacy of an AE-focused treatment, prolonged exposure (PE), in a PD population [15, 16]. PE, an evidence-based intervention for PTSD, uses exposure to reduce avoidance of both internal and external AE reminders to reduce distress and increase functioning [17–19]. Preliminary evidence suggests that PE treatment reduces symptoms of psychosis and PTSD, and does not lead to symptom exacerbation [15, 20]. However, the evidence has thus far focused almost exclusively on people with chronic psychosis rather than early phase psychosis (EPP; i.e., first 5 years of a psychotic disorder), and SM outcomes have rarely been considered. [20]. Moreover, a recent study examining unmet clinical needs in early psychosis programs across five countries highlighted the necessity of developing tailored adversity-focused treatments for people with EPP [21], suggesting a gap in both the research literature and clinical practice.

The current study aimed to examine the outcomes of an adapted PE protocol called ‘PE+’ and observe whether specifically targeting AE sequelae results in clinically significant changes in SM/psychotic symptoms. Our primary outcomes were reductions in AE sequelae, psychotic symptoms, and SM. Our secondary outcome was related to functioning; we hypothesized that social and occupational functioning would improve. We hypothesized that there would be a decrease in hopelessness and experiential avoidance (the theorized mechanisms of change of PE + treatment), as well as decreases in negative psychotic symptoms, SM, and AE sequelae. All changes were hypothesized to be maintained at follow-up 2-months post-treatment.

## Methods

### Design

The protocol of this study, which includes a detailed discussion of the methodology and intervention components, was published previously [22]. The PE + study used a multiple baseline design, which included a 2–4-week baseline measurement period functioning as a control against which to compare the measurements collected during and post-intervention [23, 24]. Participants were randomized to either the 2-week delay, 3-week delay, or 4-week delay condition. Following the baseline period, participants engaged in five three-session ‘modules’ of therapy, each focused on a different therapeutic ingredient (e.g., imaginal exposure). After each module, individuals participated in a brief assessment. Therapists were blinded to assessment results during treatment. After the sixth assessment was complete, immediately following treatment completion, there was a 2-month delay after which participants returned for two follow-up assessment appointments.

### Participants

All patients were recruited from the Nova Scotia Early Psychosis Program (NSEPP), an early intervention program for psychosis located in Halifax, Nova Scotia, Canada. The inclusion criteria for the study were as follows: (1) aged 19 to 35 years old, (2) diagnosis of a schizophrenia spectrum disorder within the last 5 years, (3) SM within the last 3 months (i.e., ‘moderate’ or higher score on the World Health Organization’s Alcohol, Smoking, and Substance Involvement Screening Test (WHO ASSIST) measure) [25], (4) have experienced 1 + AEs that continue to affect their life (i.e., affected $\:\ge\:$5 out of 10 on the Trauma and Life Events (TALE) questionnaire) [26], and (5) speaks and understands English.

Individuals could not participate if they met any of the exclusion criteria: (1) Age outside of specified age range (i.e., $\:\le\:$ 18 years, $\:\ge\:$ 36 years, ), (2) Scoring in the ‘high risk’ range for cocaine use on the WHO ASSIST, (3) does not speak English, (4) current involuntary admission or under a Community Treatment Order (due to concerns about voluntariness and capacity), (5) a diagnosed intellectual disability, or (6) current participation in an intervention to change SM or treat AE sequelae.

## Measures

The three primary outcome measures included the Trauma Symptom Checklist-40 (TSC-40) [27], Positive and Negative Syndrome Scale (PANSS) [28], and the WHO ASSIST [25]. The TSC-40 was used to establish whether adversity sequelae improved^1^. The PANSS was used to assess change in both positive and negative psychotic symptoms over time; the general psychopathology scale items were not collected to reduce participant burden. The WHO ASSIST was both an eligibility measure and outcome measure of SM, assessing risk for substance-use related harm. The Beck Hopelessness Scale (BHS) [29, 30] measured hopelessness, while the Brief Experiential Avoidance Questionnaire (BEAQ) [31] measured experiential avoidance. The Social and Occupational Functioning Assessment Questionnaire (SOFAS) [32] measured functioning, the Clinical Global Impression – Severity (CGI-S) and CGI – Improvement (CGI-I) [33] examined symptom severity, and the 8-item PTSD Checklist for DSM-5 (PCL-5) [34, 35] assessed PTSD symptoms. The TSC-40 and PCL-5 were administered at baseline, across all six assessments, and at the 2-month post-therapy follow-up assessments, while the WHO ASSIST and SOFAS were administered at the same time points except the second post-therapy follow-up assessment. The PANSS and CGI were administered at assessments one (prior to intervention start) and six (following intervention completion), and at the first follow-up assessment.

In the context of treatment fidelity, specifically treatment receipt and enactment, participants were asked to answer two questions (see Table 3) about how difficult it was to understand the information presented in sessions and how helpful this treatment was in achieving the goals they set at the beginning of treatment from “Extremely Difficult” (0) to “Extremely Easy” (10). In addition, participants were asked how easy it was to use the skills they learned in therapy from “Extremely Difficult” (0) to “Extremely Easy” (10), and how often they used these skills, ranging from “Never” (0) to “Almost Every Day or Every Day” (10). Finally, the Satisfaction with Therapy subscale of the Satisfaction with Therapy and Therapist Scale – Revised (STTS-R) [36] was administered; participants were asked seven questions about their satisfaction with treatment (e.g., treatment needs were met) ranging from “Strongly disagree” (1) to “Strongly agree” (5). Total scores ranged from 0 to 30, with higher scores indicating greater satisfaction.

### Procedure

**Fig. 1:**
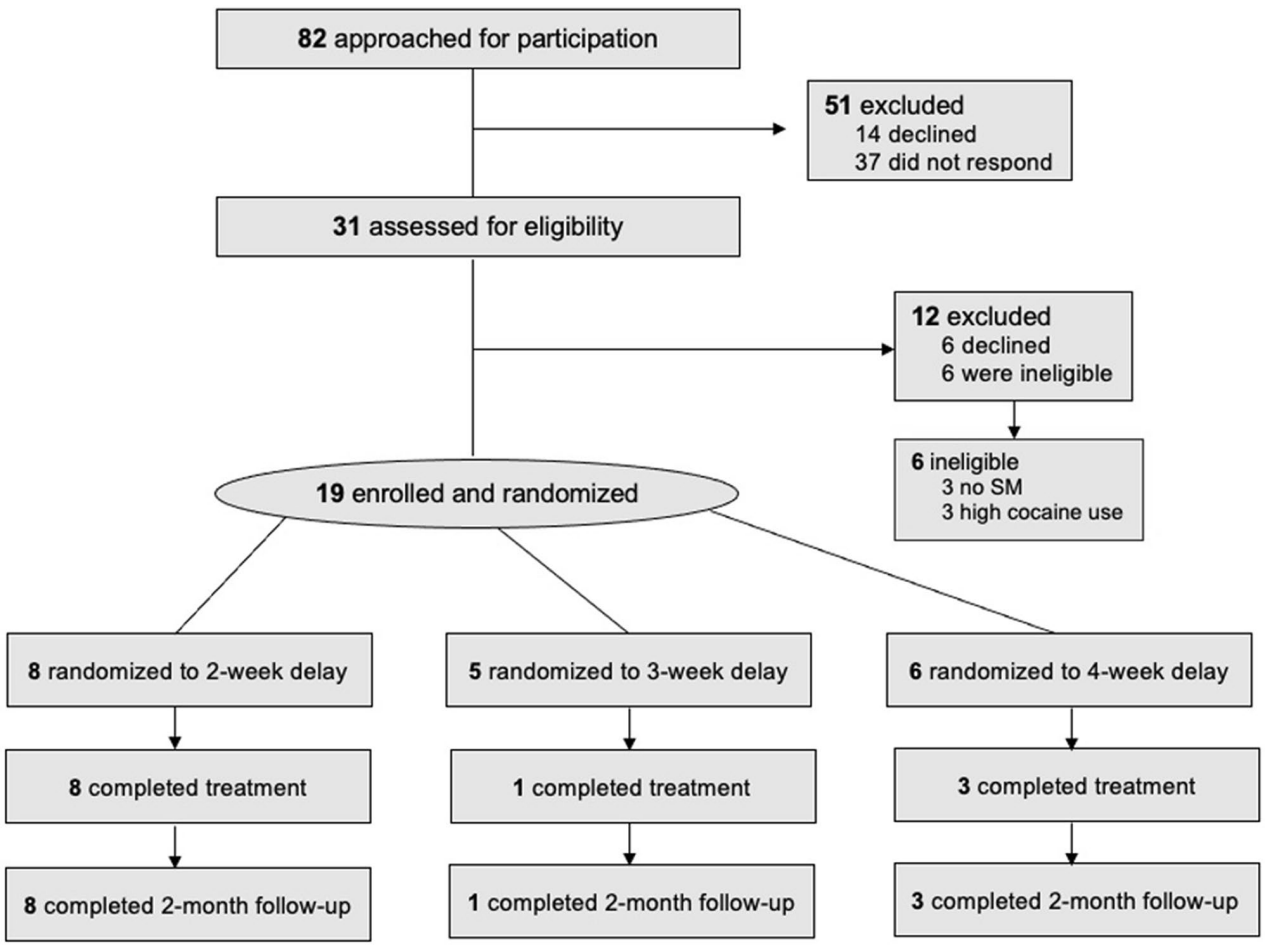
Participant recruitment information

Participants were recruited through the NSEPP from November 2021 until November 2022. Interested individuals completed an eligibility screening appointment either via telephone or in person at the clinic, after which a baseline appointment was scheduled if they were eligible. During that baseline appointment, individuals received their randomization status (i.e., 2, 3, or 4-week delay) via a sealed envelope (see Fig. 1 for participant recruitment flow). Study therapists were blinded to assessment results during treatment; there were no instances of unblinding.

### Treatment

As a part of typical clinical care at the EPP clinic, participants worked with a nurse and a psychiatrist for medication management, and had access to occupational therapy support, but they were not receiving any psychological therapy. The PE + treatment is an adaptation of the evidence-based PE therapy for PTSD. PE + involved fifteen weekly 90-minute sessions divided into five three-session modules; the first module involved psychoeducation about AEs, SM, and psychosis, and how the three influence one another, followed by a second module of adapted Dialectical Behaviour Therapy (DBT) [37] material regarding emotion identification and regulation and distress tolerance skills (i.e., temperature, intense exercise, paced breathing, paired muscle relaxation; TIPP). The next two modules were focused on imaginal and in vivo exposures, respectively; the imaginal exposures were initiated in module three (i.e., session 7 of 15) and were continued until the end of therapy, while in vivo exposures were initiated in module four (session 10 of 15) and continued until the final session. The fifth and final module consisted of relapse prevention skills. Participants were instructed to select one AE, typically the ‘worst’ event, to focus on from the start of treatment until the end. All participants were encouraged to complete therapy sessions and home practice exercises while sober.

#### Supervision and fidelity monitoring

The study therapists completed over 16 h of training prior to delivering the PE + intervention, including clinical roleplays of PE skills, and feedback on intervention skills, in addition to completing a didactic PE course. Therapists participated in weekly group supervision and ad hoc supervision as needed with a registered psychologist (AP) during the study, and they received periodic supervision from another registered psychologist (JT) regarding dissociation management.

Each therapy session was recorded and scored against the author-adapted treatment adherence checklist for that session based on treatment adherence checklists for traditional PE [38]. 10% of all sessions were randomized for inclusion in treatment adherence ratings carried out by two independent coders; sessions were randomized for inclusion by module and therapist. The adherence rating lists were conservative, and any departure from the manualized treatment (e.g., missing session agenda, insufficiently detailed recaps of previous sessions, shorter session length) was considered a lapse in adherence.

### Data Analysis

We took a stringent intent-to-treat (ITT) approach to data analysis, including all participants who were randomized in the results, regardless of whether they completed therapy. Inferential statistics were not deemed appropriate given the small sample size and our focus on clinically significant change (CSC) rather than statistically significant changes. We used the Reliable Change Index (RCI) [39] to assess change from baseline— all metrics used to establish the degree of required change can be found at clinicaltrials.gov (NCT04546178). The categories of change are CSC (i.e., full recovery), improved (i.e., partial recovery), no change (i.e., no significant change from baseline), or deterioration (i.e., significant negative change from baseline). Quantitative data pertaining to treatment fidelity (i.e., treatment enactment, receipt) were gathered from participants and descriptives were computed for the quantitative data, along with qualitative feedback about participants’ experiences in treatment.

## Results

### Participants

Nineteen individuals with a psychotic spectrum disorder participated in the PE + study (see Table 1). The majority of participants were white and had a diagnosis of schizophrenia, and 42% of the sample were members of the 2SLGBTQ + community given their gender or sexual orientatio-+n. Participants commonly misused cannabis (100%) and alcohol (53%), although participants used 2.3 substances on average (SD = 1.3, range 1–6). SM at baseline was as follows: cocaine (37%), hallucinogens (26%), sedatives (26%), amphetamines (16%), inhalants (5%), and other substances (5%). Inhalants and opioids will be grouped under ‘other substances’ given their low frequency throughout the study. Participants experienced a multitude of different AEs; the AEs most commonly experienced by participants, by category, was as follows: bullying and being put down/humiliated by someone close to the participant (84%; interpersonal AEs), terrifying psychotic symptoms (79%; psychosis-related AEs), and experiencing illness/disaster (e.g., house fire; 42%; non-interpersonal AEs).

**Table 1:** Participant demographics at baseline

	Participants *N* = 19
**Age**, *M* (SD)	24.9 (3.6)
**Race**,** %**	
Black	10.5% (2)
Multiracial	10.5% (2)
White	74% (14)
Unknown	5% (1)
**Gender**, %	
Non-binary	26.3% (5)
Man	52.6% (10)
Woman	21.1% (4)
**Sexual orientation**, %	
Asexual	5.3% (1)
Bisexual	5.3% (1)
Gay/Lesbian	10.5% (2)
Heterosexual	57.9% (11)
Pansexual	15.8% (3)
Queer	5.2% (1)
**Number of months at NSEPP**, *M* (SD)	24 (23)
**Diagnosis**, % (n)	
Brief Psychotic disorder	5.2% (1)
Psychotic disorder not otherwise specified (NOS)	15.8% (3)
Schizophrenia	57.9% (11)
Schizoaffective	15.8% (3)
Substance-induced psychotic disorder	5.3% (1)
**Adverse events**, *M* (SD)	
Lifetime AEs	11.8 (2.5)
Interpersonal AEs	7.2 (2.9)
Psychosis-related AEs	2.5 (0.9)
Non-interpersonal AEs	1.4 (0.7)

Note: NSEPP = Nova Scotia Early Psychosis Program (NSEPP); AEs = Adverse Events

Four participants dropped out during the baseline period (i.e., pre-therapy), and three participants dropped out during treatment. No serious adverse events as a result of study participation were reported. Five participants reported dropping out due to changes in life circumstances (e.g., moving, change in work hours), one participant did not want to discuss their AE, and one did not disclose their reasons. Of those who dropped out during treatment, two participants completed at least half of treatment, while the other participant completed only six sessions before dropping out. The sample of treatment starters was *n* = 15, and there were *n* = 12 treatment completers.

### Symptom outcomes

**Fig. 2:**
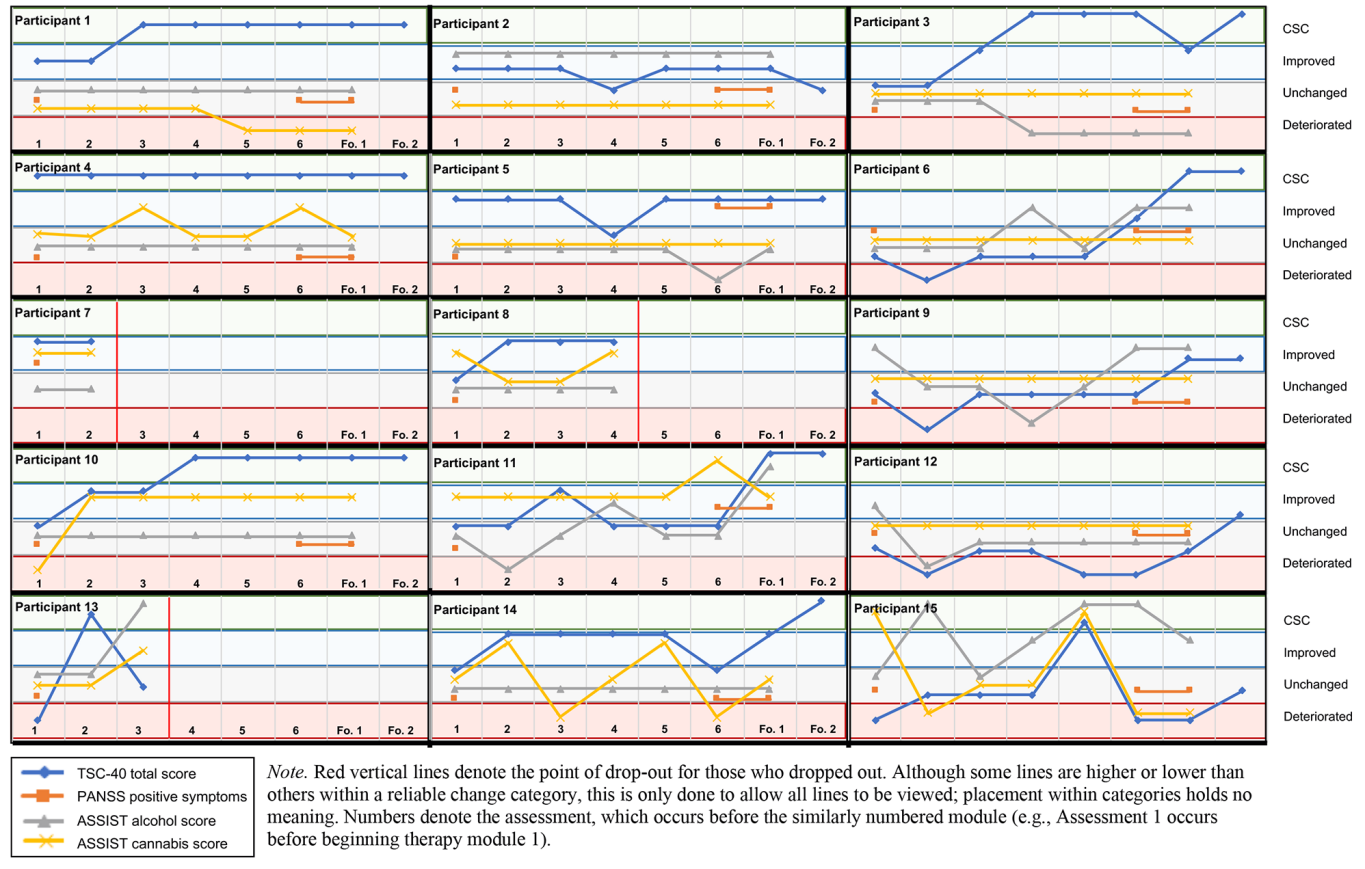
RCI scores for primary outcomes (TSC-40 total, alcohol, cannabis) and positive psychotic symptoms

**Fig. 3:**
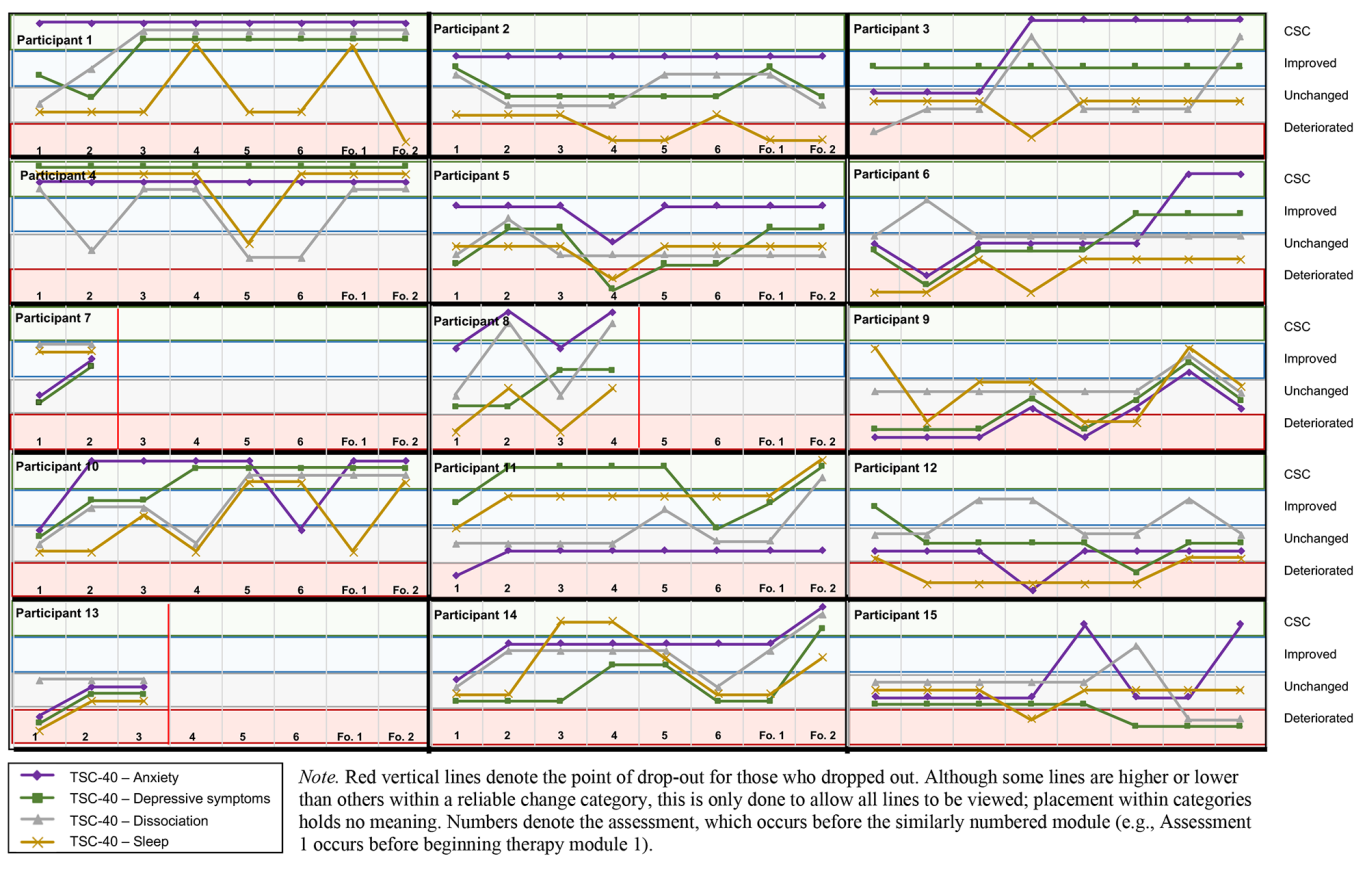
RCIs for TSC-40 subscales (anxiety, depressive symptoms, dissociation, sleep) across PE+ treatment

Table 2 lists the baseline, post-therapy, and follow-up symptom measurements. When reviewing individual changes using the RCI (see Fig. 2), a pattern emerges. Most participants (83%) experienced an amelioration in overall AE-related psychopathology (improvement or CSC) on the TSC-40 by follow-up (Δmeans = 20.9), with 58% achieving CSC. The course of improvement was often one involving temporary improvements or deteriorations on an upward trajectory. Symptoms appeared to consistently improve after Module 3, the imaginal exposure-focused module. When examining specific domains of the TSC-40 results (see Fig. 3), half or more of the participants achieved CSC on dissociation and anxiety, and just under 50% achieved CSC on the depressive symptom domain. However, the courses across participants varied significantly–dissociation had a similar pattern to the TSC-40 total score, increasing and returning to baseline before increasing again, whereas anxiety and depressive symptoms stayed consistent once an amelioration had occurred, although there were more early deteriorations from baseline observed at assessment 1 compared to dissociation and TSC-40 total scores. Sleep appeared to improve little, deteriorating or remaining unchanged for most participants. The results of the abbreviated PCL-5 also suggested improvement; 58.3% of participants achieved CSC by follow-up. Participants who experienced improvements in PCL-5 scores typically maintained those improvements over time. The PANSS positive symptoms (Δmeans = 5.8) improved for two participants (16.7%) and that change was maintained at follow-up, but there were no changes to negative psychotic symptoms for any participants. In terms of the proposed mechanisms, only one participant experiencing improvement to hopelessness (8%), although there was clinically significant improvement to experiential avoidance for a third of treatment completers (*n* = 4), despite somewhat limited change to group means from baseline (Δmeans = 8.9).

**Table 2:** Mean clinical scores at baseline, post-treatment, and at follow-up

	Baseline *N* = 19 M (SD)	Post-therapy *N* = 12 M (SD)	2 months follow-up *N* = 12 M (SD)
**PCL-5 (8-item)**	17.8 (5.9)	10.1 (6.4)	8.5 (6.1)
**BEAQ**	54.7 (10.2)	49.1 (12.5)	50.5 (11.6)
**BHS**	7.1 (5.1)	5 (4.6)	4.5 (4.6)
**TSC-40 total score**	45.2 (17.5)	31.1 (20.8)	24.7 (16.4)
TSC-40 - Anxiety	8.5 (5.0)	5.1 (4.2)	4.2 (3.4)
TSC-40 - Depression	10.8 (3.9)	7.3 (5.8)	5.7 (4.2)
TSC-40 - Dissociation	9.1 (3.7)	6.8 (4)	5.4 (2.6)
TSC-40 - Sleep	7.3 (4.4)	5.6 (4.6)	4.6 (4.2)
**PANSS positive symptoms**	18.1 (5.6)	12.8 (0.3)	12.2 (2.8)
**PANSS negative symptoms**	14.8 (3.4)	13.3 (3.7)	15.2 (6.2)
**SOFAS**	57 (9.3)	67.7 (13.1)	69.5 (13.6)
**CGI-S**	3.8 (0.9)	3.2 (0.9)	3.5 (1.2)
**WHO ASSIST**			
Alcohol	12.3 (7.2)	11.5 (8.8)	10 (8.9)
Cannabis	21.8 (9.8)	19.5 (10.7)	3.5 (9)
Cocaine	9 (7.6)	6.5 (0.5)	3.5 (0.5)
Amphetamines	5.8 (4.3)	6 (3)	3 (0)
Inhalants	6 (0)	-	-
Sedatives	5.3 (2.2)	4.5 (1.5)	4 (2)
Hallucinogens	6.7 (4.1)	6 (0)	2.7 (0.9)
Opioids	3 (0)	-	2 (0)
Other substances	6 (0)	-	2 (0)

*Note*: PCL-5 = PTSD Checklist 5; BEAQ = Brief Experiential Avoidance Questionnaire; BHS = Beck Hopelessness Scale; TSC-40 = Trauma Checklist 40; PANSS = Positive and Negative Syndrome Scale; SOFAS = Social and Occupational Functioning Assessment Scale; CGI-S = Clinical Global Impression – Severity scale; WHO ASSIST = Alcohol Smoking, Substance Involvement Screening Test. The WHO ASSIST scores are only calculated for those who use each substance. A dash is present if that substance was not used by any participants

Group-level alcohol scores did not appear to vary substantially at follow-up (Δmeans = 1.4), but on an individual level, four participants experienced ameliorations to their alcohol use by follow-up (50%), although only one met criteria for CSC (12.5%). However, four participants experienced increases in alcohol use scores (deteriorations) throughout the study, although these increases were temporary for three participants; the fourth maintained the increase at follow-up. Similarly, four participants experienced increases in cannabis scores (26.7%); two participants maintained the increased use by the follow-up period (16.7%), one returned to their baseline score (8.3%), and one experienced improvement (8.3%). The cannabis group mean marginally shifted (Δmeans = 2.5). Although not used as frequently as cannabis or alcohol, there was an amelioration to hallucinogen use by follow-up for 71% of the sample of treatment completers who used hallucinogens (*n* = 5), while no changes were observed to cocaine or sedative scores at any time, and a brief amelioration for one participant using amphetamines before returning to their baseline score.

### Treatment fidelity and therapeutic alliance

Inter-rater reliability for treatment videos was strong; kappas ranged from 0.83 to 0.93 across modules (*M* = 0.88, SD = 0.04). The overall mean adherence rating was 82% (SD = 15%), considered adherent [40]. Table 3 lists all quantitative treatment fidelity ratings. Participants reported that the information presented in treatment was easy to understand and apply to their everyday lives. The average frequency of skill use (e.g., exposure) was reported to be moderate, ranging from occasional use to every day, suggesting appropriate levels of treatment enactment overall. At follow-up, participants reported a similar frequency of skill use, with only one participant reporting a reduction in skill use frequency since ending treatment. Completion of between-session tasks (i.e., participant homework) was monitored throughout treatment to assess treatment enactment. Participants at least partially completed homework in 89% of sessions, and 50% of all sessions included completion of all homework. Differences between therapists on ratings of overall therapeutic alliance with clients were minor [range: 36.2–38.3], as were differences in ratings between modules [range: 35.2–38.6]. Overall, ratings of therapeutic alliance were high, suggesting a strong alliance between therapists and participants in this study.

**Table 3:** Participant receipt and enactment and participant opinions about treatment (*n* = 12)

	Mean (SD)
*Post-treatment receipt and enactment*	
How easy was it to understand the information presented in sessions?	8.6 (1.6)
How easy was it to use the skills you learned in the therapy sessions in your day-to-day life?	7.0 (2.0)
On average, how often did you use the skills you learned?	6.6 (2)
How helpful was this therapy in helping you to achieve the goals you set at the beginning of treatment?	7.5 (1.2)
Overall, how difficult was it to do this therapy?	6.4 (2.2)
*2-month follow-up receipt and enactment*	
Since ending therapy 2 months ago, how easy has it been to use the skills you learned in the therapy sessions in your day-to-day life?	6.4 (2.2)
Since ending therapy 2 months ago, how often have you used the skills you learned?	6.6 (1.2)
*Satisfaction with Therapy*	
I am satisfied with the quality of therapy I received	4.6 (0.5)
My needs were met by the therapy	4.5 (0.5)
I would recommend this therapy to a friend if they needed it	4.4 (0.7)
I would come back to the clinic for this therapy if I needed help	4.5 (0.7)
I am now able to deal more effectively with my problems	4.0 (0.9)
I was able to focus on what was of real concern to me in therapy	4.3 (0.9)

*Note*: These items were administered to the completer sample (*n* = 12). Receipt and enactment ratings were rated from 0 to 10 and participant ratings of treatment ranged from 0 to 5; higher ratings indicated greater frequency of skill use, ease of understanding, agreement, etc

### Participant feedback

Feedback was quite consistent across participants. When asked about the most helpful element of treatment, several treatment completers noted that exposure, although difficult, was the most helpful aspect of treatment (41.7%; 5/12). However, participants also remarked that coping strategies alone (DBT skills, e.g., check the facts, TIPP) [37] (25%; 3/12) and both exposure and coping strategies (25%; 3/12) were the most helpful treatment components. One participant did not respond. It is noteworthy that during an open-ended request for feedback on the therapist, therapy, and research study experience, 50% of treatment completers (6/12) remarked on the vital role that their therapist played in treatment, highlighting how helpful it was that their therapist had a nonjudgmental approach and made efforts to understand their experience. Table 3 describes the quantitative study feedback; overall, participants were very satisfied with the PE + treatment.

When asked how this treatment approach could be adapted to better meet the needs of those who may need it in future, treatment completers consistently mentioned increasing the number of sessions (75%; 9/12). Some participants (16.7%; 2/12) wanted more emphasis on psychotic symptoms, given the links between their AEs and psychosis, as well as additional skills to cope with hallucinations. These participants discussed the importance of being able to discuss the interconnections between their AEs and their psychotic symptoms, especially when the AE occurred during or was a psychotic episode.

## Discussion

Primary outcomes of PE + included improvements across most domains of AEs sequelae (i.e., dissociation, depressive symptoms, anxiety) including PTSD symptoms, no changes to negative psychotic symptoms, and 16.7% (2/12) participants experienced clinically significant improvements in substance use by follow-up. However, 40% (6/15) did achieve temporary improvements in SM during the study, although they were not maintained by follow-up. Several participants had substance use deteriorations, although no deteriorations in psychotic symptoms were observed. Our hypotheses were not supported with regards to improvements to psychotic symptoms or substance use, but improvements in AE sequelae (i.e., anxiety, dissociation, depressive symptoms, PTSD symptoms) by follow-up did support hypotheses.

In terms of secondary outcomes, treatment starters experienced some improvements in experiential avoidance (33%) and functioning (46.7%), although only 33% of the sample maintained functioning improvements at follow-up. Few changes to hopelessness were observed (6.7%); less than half the sample experienced significant change, meaning that hypotheses for experiential avoidance, hopelessness, and functioning were not supported. Only two participants (13.3%) experienced improvements in positive psychotic symptoms by follow-up. Participants were satisfied with the quality of treatment and found the PE + treatment helpful to achieve their goals, which aligns with previous participant experiences with adversity-focused treatments [41]. Participants noted that exposure and emotion-focused skill-building were the most helpful elements of treatment.

Several other adversity-focused treatment trials also observed improvements to AE-related sequelae consistent with our results. Keen and colleagues [42] reported that 63% of their participants with psychotic symptoms achieved CSC to AE sequelae during their longer protocol (median number of sessions = 41), while van den Berg and colleagues [15] found that more than 56% of their participants with chronic psychosis no longer met criteria for PTSD by the 6-month follow-up assessment after the end of their 8-session treatment. It was unclear whether the improvements to AEs sequelae found in these previous studies would translate to an EPP population, however our findings suggest that similar improvements can be expected. In contrast, there were fewer improvements to positive psychotic symptoms in the PE + study than in other studies, where small effects were found in pre-post analyses, although negative symptoms remained unchanged in multiple trials [43]. However, no other study focused exclusively on an EPP sample, instead using predominately chronic or mixed-duration samples who were typically at least 10–15 years older than those usually in EPP. It is noteworthy that no deteriorations in psychotic symptoms were observed during the PE + trial. Although previous adversity-focused trials among people with chronic psychosis similarly observed few psychotic symptom exacerbations [20], a consistent barrier to the delivery of adversity-focused treatments among patients with psychotic disorders is clinician fear of psychotic symptom exacerbation [9]. The PE + trial results may provide clinicians with more confidence that adversity-focused treatments appear to be safe for people with EPP.

To our knowledge, this is the first adversity-focused treatment trial for people with EPP, and the first to integrate and measure SM over time. Current clinical guidelines for addressing AEs with people with psychotic disorders [14] suggest integrated treatment with one clinician addressing psychosis and AEs sequelae simultaneously. We suggest extending these guidelines to include substance use challenges as well and adopting an integrated approach to treatment for psychosis, SM, and AEs. We hypothesized that by targeting AE-related symptoms, we may indirectly improve all symptom domains (e.g., psychosis, SM) given that SM may function as a coping mechanism for AE sequelae, as suggested by the stress and coping model of SM, and AE sequelae may be maintaining psychotic symptoms through shared mechanisms (e.g., dissociation). Our results do not appear to support this hypothesis and SM deteriorated in several cases, suggesting an alternative approach may be needed. Rather than targeting one domain, it may be more helpful to take a fully integrated approach, incorporating psychosis-specific treatment strategies from Cognitive Behavioural Therapy for Psychosis (CBTp) [44], and integrating SM strategies with PE+, in a similar way as with the Concurrent Treatment of PTSD and SUDs using Prolonged Exposure (COPE) [45] protocol. A more integrated approach may allow a greater focus on treating the links that may be maintaining these challenges (e.g., cannabis use to reduce anxiety caused by PTSD which worsens auditory hallucinations).

In our sample, SM was more frequently unchanged or worsened than improved, suggesting even more direct targeting of substance use may be required to change use in a group with psychosis and a history of AEs, especially because PE treatment in non-psychosis populations did not result in SM increases [46]. Moreover, the literature on substance use motives (e.g., to cope, improve mood) related to AEs [47, 48] suggests that a greater understanding of the function of substance use within the AE-SM-EPP relationship may allow for more explicit targeting within future interventions.

Participants also expressed benefiting from the coping strategies module, which is a stabilization module, suggesting its acceptability among participants. However, other trials have found positive effects without stabilization. Brand and colleagues [49] suggested that the acceptability and efficacy of a stabilization phase should be examined; although acceptable to our participants, no changes to psychopathology scores were observed immediately following this phase.

A significant strength of the PE + trial is the comprehensive treatment fidelity approach. We created and reported a comprehensive treatment fidelity plan, including a treatment manual, therapist training, video coding for adherence, and measures of both treatment receipt and enactment. This robust approach is not always used [50, 51]. Although comprehensive, we suggest future studies explore the psychometrics of our adapted fidelity checklists. In addition, we tested the PE +  protocol embedded within an early psychosis program, meaning that it was tested in the environment in which it would be delivered in the future, which increases the ecological validity of PE+. The two limitations of these results involve sample size and limited diversity of race and culture (although there was substantial sample diversity on sexual orientation and gender identity). Given the preliminary nature of this study, a small sample was appropriate; however, generalizing these results to a larger sample may present challenges, especially if the program structure differs from the standard approach to care detailed above. Moreover, the racial and cultural diversity of this sample was limited; few ethnicities were represented in this sample and the majority of participants reported that their race was white. This may make generalizing PE + results to other races and cultures more difficult.

## Conclusions and future directions

In conclusion, PE + treatment appeared to be helpful for AEs sequelae but was less effective at improving psychotic symptoms and substance use. However, importantly, psychotic symptoms did not worsen despite the exposure component. Participants found PE + to be helpful and noted that exposure and coping skills were the most helpful elements of treatment. A few participants believed that treatment did not focus enough on psychotic symptoms. Further work needs to be done to establish an effective, integrated treatment capable of addressing the links between psychotic symptoms, SM, and AEs sequelae. Integrating more substance use treatment strategies (e.g., coping with cravings) and increasing the focus on treating psychotic symptoms in PE + may be a more beneficial approach. Such a ‘PE++’ approach could be developed from the current intervention and be piloted before bringing this expanded adversity-focused approach to a full randomized controlled trial (RCT) comparing to standard PE, PE+, and a TAU condition. Moreover, future studies should explore a longer treatment duration; AE-focused treatment studies with people with PDs have consistently found that protocols using 8–15 sessions are too short. Lastly, future adversity-focused treatment trials should include adolescents in their early psychosis samples to understand whether adversity sequelae improvements observed with young adults will generalize to a younger age group.

## Data Availability

The datasets generated and analyzed during the current study are not publicly available as participant consent for publication in raw form was not obtained, but data are available from the corresponding author upon reasonable request.

## Abbreviations

2SLGBTQ +: Two-spirit, lesbian, gay, bisexual, transgender, queer + individuals
AE(s): Adverse event(s)/adversity
ASSIST: Alcohol, smoking, and substance involvement screening test
BEAQ: Brief experiential avoidance questionnaire
BHS: Beck hopelessness scale
CBTp: Cognitive behavioural therapy for psychosis
CGI-I: Clinical global impression – Improvement of illness
CGI-S: Clinical global impression – Severity of illness
COPE: Concurrent treatment of PTSD and SUDs using prolonged exposure
CSC: Clinically significant change
DBT: Dialectical behaviour therapy
EPP: Early phase psychosis
ITT: Intent-to-treat
NSEPP: Nova scotia early psychosis program
PANSS: Positive and negative syndrome scale
PCL-5: Posttraumatic stress disorder checklist-5
PD: Psychotic disorder(s)
PE: Prolonged exposure therapy
PE +: Adapted prolonged exposure therapy
PTSD: Posttraumatic stress disorder
RCI: Reliable change index
RCT: Randomized controlled trial
STTS-R: Satisfaction with therapy and therapist scale - Revised
SD: Standard deviation
SM: Substance misuse
SOFAS: Social and occupational functioning assessment scale
TALE: Trauma and life events checklist
TIPP: Temperature, intense exercise, paced breathing, progressive muscle relaxation
TSC-40: Trauma symptom checklist - 40

## Footnotes

1 Although an abbreviated 8-item version of the PCL-5 was also used within this study and results are reported below, the PCL-5 was considered a secondary measure because it is specific to PTSD symptoms, whereas the TSC-40 is a broadband measure assessing various domains of psychopathology relevant to AEs (e.g., anxiety, depression).
